# Flat nodular lymphoid hyperplasia resembling “apple tree branches” in the colon

**DOI:** 10.1055/a-2086-2240

**Published:** 2023-06-12

**Authors:** Stefan Mitev, Evelina G. Atanasova, Diana Kyoseva

**Affiliations:** 1 Gastroenterology Clinic, University Hospital St Ivan Rilski, Sofia, Bulgaria; 2 Department of General and Clinical Pathology, University Hospital Alexandrovska, Sofia Medical University, Sofia, Bulgaria

A 19-year-old woman with a history of adjustment disorder presented with abdominal pain, bloating, and chronic diarrhea. Colonoscopy revealed multiple flat whitish areas, less than 3 mm in diameter, diffusely distributed throughout the entire colon and rectum. These findings were prominent under narrow-band imaging (NBI) ( Fig. 1 a ) and more difficult to distinguish with white-light imaging ( Fig. 1 b ). The flat areas and the surrounding superficial vessels were described in the report as resembling “apple tree branches” ( Video 1 ). Inspection of the terminal ileum was unremarkable, and esophagogastroduodenoscopy was unrevealing. Histologic examination of the colonic biopsy specimens showed nodules of benign lymphoid tissue with enlarged germinal centers and an intact mantle zone, consistent with a diagnosis of nodular lymphoid hyperplasia (NLH) ( Fig. 2 ). Laboratory investigations and fecal calprotectin were within normal limits. Stool pathogen testing was negative. The patient was diagnosed with irritable bowel syndrome (IBS) and diffuse flat NLH.

**Fig. 1:**
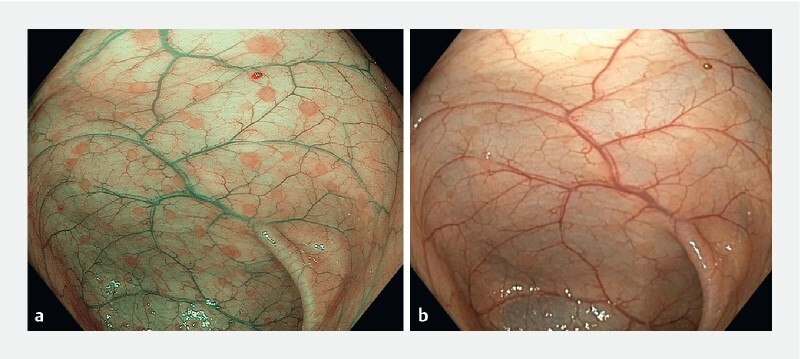
Endoscopic images showing flat nodular lymphoid hyperplasia under: **a** narrow-band imaging; **b** white-light imaging.

**Video 1** Flat nodular lymphoid hyperplasia resembling “apple tree branches” is seen in the colon during colonoscopy.


**Fig. 2:**
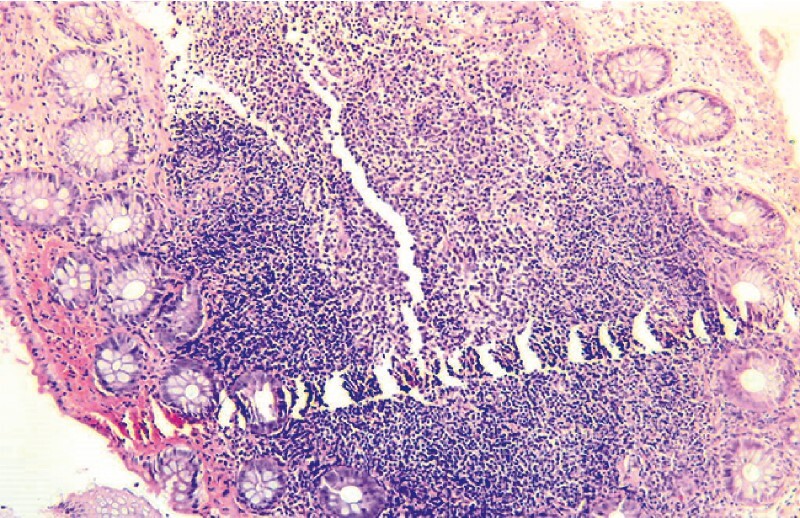
Histologic appearance showing a nodule of benign lymphoid tissue with an enlarged germinal center and an intact mantle zone.

A prior study reported that functional gastrointestinal disorders, now called disorders of gut–brain interaction (DGBI), were diagnosed in 63 % of the symptomatic patients with NLH 1 , with 91 % of these patients fulfilling the Rome III criteria for IBS, mainly IBS-D subtype. NLH has also been found in patients with organic diseases, in particular inflammatory bowel disease, parasitic and bacterial infections, common variable immunodeficiency, selective IgA deficiency syndrome, celiac disease, human immunodeficiency virus (HIV), food hypersensitivity, diverticular disease, microscopic colitis, ischemic colitis, and colorectal carcinoma.

During endoscopy, the lymphoid follicles may be seen as flat, slightly elevated, or polypoid areas. Usually, their size does not exceed several millimeters. Sometimes, the lymphoid follicles have a reddish outline, which has been referred to as the “red ring sign.” Currently, there are no clear guidelines for the follow-up and surveillance of patients with NLH 2 . This case illustrates that NBI is a useful modality for the diagnosis of flat NLH.

Endoscopy_UCTN_Code_CCL_1AD_2AC

## Notes

Endoscopy E-Videos

https://eref.thieme.de/e-videos
*E-Videos* is an open access online section of the journal *Endoscopy* , reporting on interesting cases and new techniques in gastroenterological endoscopy. All papers include a high-quality video and are published with a Creative Commons CC-BY license. Endoscopy E-Videos qualify for HINARI discounts and waivers and eligibility is automatically checked during the submission process. We grant 100% waivers to articles whose corresponding authors are based in Group A countries and 50% waivers to those who are based in Group B countries as classified by Research4Life (see: https://www.research4life.org/access/eligibility/ ). This section has its own submission website at https://mc.manuscriptcentral.com/e-videos
